# Recombinational landscape of porcine X chromosome and individual variation in female meiotic recombination associated with haplotypes of Chinese pigs

**DOI:** 10.1186/1471-2164-11-159

**Published:** 2010-03-09

**Authors:** Junwu Ma, Nathalie Iannuccelli, Yanyu Duan, Weibing Huang, Beili Guo, Juliette Riquet, Lusheng Huang, Denis Milan

**Affiliations:** 1 Laboratoire de Génétique Cellulaire, INRA, BP52627, 31326 Castanet-Tolosan, France; 2 Key Laboratory for Animal Biotechnology of Jiangxi Province and the Ministry of Agriculture of China, Jiangxi Agricultural University, Nanchang 330045, China

## Abstract

**Background:**

Variations in recombination fraction (θ) among chromosomal regions, individuals and families have been observed and have an important impact on quantitative trait loci (QTL) mapping studies. Such variations on porcine chromosome X (SSC-X) and on other mammalian chromosome X are rarely explored. The emerging assembly of pig sequence provides exact physical location of many markers, facilitating the study of a fine-scale recombination landscape of the pig genome by comparing a clone-based physical map to a genetic map. Using large offspring of F_1 _females from two large-scale resource populations (Large White ♂ × Chinese Meishan ♀, and White Duroc ♂ × Chinese Erhualian ♀), we were able to evaluate the heterogeneity in θ for a specific interval among individual F_1 _females.

**Results:**

Alignments between the cytogenetic map, radiation hybrid (RH) map, genetic maps and clone map of SSC-X with the physical map of human chromosome X (HSA-X) are presented. The most likely order of 60 markers on SSC-X is inferred. The average recombination rate across SSC-X is of ~1.27 cM/Mb. However, almost no recombination occurred in a large region of ~31 Mb extending from the centromere to Xq21, whereas in the surrounding regions and in the Xq telomeric region a recombination rate of 2.8-3.3 cM/Mb was observed, more than twice the chromosome-wide average rate. Significant differences in θ among F_1 _females within each population were observed for several chromosomal intervals. The largest variation was observed in both populations in the interval *UMNP71-SW1943*, or more precisely in the subinterval *UMNP891-UMNP93*. The individual variation in θ over this subinterval was found associated with F_1 _females' maternal haplotypes (Chinese pig haplotypes) and independent of paternal haplotype (European pig haplotypes). The θ between *UMNP891 *and *UMNP93 *for haplotype 1122 and 4311 differed by more than fourteen-fold (10.3% vs. 0.7%).

**Conclusions:**

This study reveals marked regional, individual and haplotype-specific differences in recombination rate on SSC-X. Lack of recombination in such a large region makes it impossible to narrow QTL interval using traditional fine-mapping approaches. The relationship between recombination variation and haplotype polymorphism is shown for the first time in pigs.

## Background

Recombination rate can vary dramatically among species, among chromosomes within species, among regions within chromosomes, and among individuals and families within regions in mammals [1]. These variations may have important consequences for the accuracy of marker assisted selection, genetic diagnosis and for the success of positional cloning or positional identification of disease gene and quantitative trait loci (QTL) [2]. Noor et al. [3] demonstrated that variance in recombination rate across a genome can cause systematic biases in the interpretation of mapping results.

The mammalian X chromosome contains a disproportionately high number of genes and QTL influencing development, female/male fertility, reproduction and diseases (OMIM, http://www.ncbi.nlm.nih.gov/sites/entrez?db=omim; OMIA, http://omia.angis.org.au/[4,5]), notably in pigs http://www.animalgenome.org/cgi-bin/QTLdb/SS/draw_chromap?chromos=x. For example, Milan et al. [6] detected a few major QTL on the porcine X chromosome (SSC-X) that explained more than 40% of the F_2 _phenotypic variation for backfat weight and muscle contents in a Large White × Meishan pig resource population. Identification of causal genes underlying these QTL could have great economic significance for the pig industry. It could also provide very valuable insights into the genetic regulation of fat deposition in mammals. However, this purpose can hardly be achieved because, so far, little is known about between-region, individual and family variation in recombination that occurs on SSC-X.

The increasing wealth of genetic and physical mapping data makes it possible to detail precisely patterns of recombination on SSC-X. The larger the family, the more reliable is the estimate of genetic distance. Physical distance between markers can be estimated by radiation-hybrid (RH) mapping, while its exact value will be determined on the complete genome sequence [7].

Sperm typing studies have revealed significant variations between individual human males [8] and between individual bulls [9,10] in the fine-scale rate of crossing over. Such studies, however, can only be performed on male recombination, so the only access to fine-scale patterns of female recombination could only be obtained through a classical analysis of families [11]. A valuable case for such a study is the X chromosome, because it is only there that female recombination occurs in the absence of male recombination [12]. So far, pedigree studies have identified variation in the global recombination rate (total genetic-map length) among mothers and have shown that the variation is heritable, suggesting that there are some underlying components determined by both genetic and environment factors that affect maternal recombination rates [13,14]. However, no study to date has documented the variation in fine-scale rate among females, due to the limited number of children per mother [15]. Fortunately, the higher fertility of the pig compared to other mammalian species effectively increases the sample size of meiotic products that can be obtained from individual females, permitting direct comparisons between animals.

Heterogeneity in recombination rate between the same loci on different linkage maps may represent genetic variation in some aspects of the meiotic recombination machinery among individuals of different mapping pedigrees. This variation may be multifactorial, including differences in sex, genetic background, haplotype, age, recombination-promoting sequences, chromosome size, sequence homology, and sites for initiation of chromosome pairing [10]. For instance, the presence of recombination hotspots within mouse major histocompatibiltiy complex (MHC) have been detected in some specific MHC haplotypes [16,17], therefore the frequency of recombination in this region can vary among individuals or strains carrying different haplotypes.

Previously, Large White ♂ × Chinese Meishan ♀ (LW × MS) and White Duroc ♂ × Chinese Erhualian ♀ (WD × ER) F_2 _intercross populations were established by INRA in France [18] and Jiangxi Agricultural University (JXAU) in China [19], respectively. Based on the large number of offspring (and thus meioses) per F_1 _sow, we were able to estimate recombination rate over X chromosome intervals by comparing genetic and physical maps. Then we studied the degree of heterogeneity in broad-scale and fine-scale recombination rate between individual F_1 _females, and finally compare these relationships to differences in maternal and paternal haplotypes of F_1 _females transmitted from Chinese and European F_0 _founders.

## Results

### Estimation of the most likely marker order and regional variation in recombination rate along SSC-X based on comparison of maps

We used a total number of 60 markers in this study. Among them 33 were developed for this study (see "Material and Methods" and Additional File 1 - Table S1). In addition to 16 previous ones, 19 new markers were mapped on the INRA-University of Minnesota porcine (IMpRH) radiation hybrid panel. Twenty-eight and 27 markers were mapped using the INRA and JXAU families respectively; 21 of these were mapped using both pedigrees. The sequence of 54 markers matched sequences from Sscrofa8 assembly available on the Ensembl website, and 50 marker-containing BAC clones were anchored on the human physical map. Table 1 shows the positions of markers on the porcine cytogenetic map, clone map, genetic map and RH map and on the human physical map. The consensus order was chosen as the order consistent with the majority of the maps. There is a very high conservation of marker order between the human physical map and the pig clone map, except for two small segments (*UMNP71-UMNP1218 *and *ACSL4-MCST96O22*). For these two segments, both the current swine genetic map and the RH map support the marker orders determined using the human physical map rather than those using the pig clone map, suggesting possible mistakes in the pig sequence assembly, unless chromosome inversions occurred in the DNA of the Duroc pig selected for sequencing.

**Table 1:** Locations of all markers on the different porcine maps and comparison with those on the human physical map

Ord.^1^	Marker name^2^	SSC-X cyto-genetic map	Blast matched clones on pig genome^3^	IMpRH map_Carthagene (cR)^4^	IMpRH map_INRA2005 (cR)^5^	SSC-X clone map (Mb)^6^	HSA-X physical map (Mb)^7^	INRA genetic map (Kosabi; cM)	JXAU genetic map (Kosabi; cM)^8^	USDA-MARC genetic map at NCBI (cM)^9^
1	SW949	Xp24/Yp	CH242-231E5	58	2126	0			0	0
2	**SW980**	Xp24-23	CH242-336E9	285	1861	7.65	11.38	0	16	11.9
3	**SW1903**	Xp21	CH242-273O11	523	1588	21.28	25.47	23.7	46.6	33
4	**SW2456**	Xp12	CH242-31B7	864	1345	38.46	42.14	46	65.5	55.4
5	**UMNP1174**	Xp11.2	CH242-69I19	976	1253	42.25	47.22	56.5	76.1	
6	SW2476	Xp11.2	CH242-24N13	985	1250	42.70	48.32			**77.6**
7	**SWR1861**			992	1211			59.5	78.7	65.7
8	UMNP448		CH242-147G7	1027	1200	43.78	50.42			
9	BE102J23.0003R1		CH242-102J23			44.30	50.94			
10	**SW259**	Near centro-mere	CH242-225C1	1293	1045	54.21	63.35	62.7	79.9	**74.4**
11	MCSE3F14		CH242-3F14	1301	1093	55.27	65.30			
12	BE145J20.0597R1	Xq12	CH242-145J20			58.94	69.06			
13	MCST2J13	Xq13	PigE-2J13	1480		63.85	74.74			
14	**SW1994**	Xq13	CH242-123K13	1547	1007	67.72	79.92	62.8	80.1	**74.4**
15	MCSE58H4	Xq13	CH242-58H4	1596	983	70.38	83.46			
16	BE8B11.0679Y1	Xq21	CH242-8B11			77.85	97.31			
17	MCSE65L7	Xq21	CH242-65L7	1768	932	82.80	91.66			
18	**UMNP71**	Xq21	CH242-203F13	1797	905	**84.83**	93.18	62.8	80.2	
19	UMNP374	Xq21	CH242-74J23	1840	840	**77.38**	97.00			
20	**UMNP1218**	Xq21	CH242-166I17	1884	800	**80.02**	99.84	62.8	80.4	
21	BE497I6FB48R	Xq22	CH242-497I6			88.52	103.76			
22	BE218F2FB67K	Xq22	CH242-218F2			89.33	104.46	63.2		
23	*SERPINA7E2B114M*	Xq22	CH242-427M6			89.90	105.02		80.9	
24	BE151D17.0014Y1	Xq22	CH242-151D17			90.42	105.88			
25	**SW1426**	Xq22	CH242-264N4	1991	710	91.73	107.08	65.7	83.5	**71.7**
26	BE32D24.0584R1	Xq22	CH242-32D24			91.73	107.03			
27	BE276J1FB107R	Xq22	CH242-276J1		700	91.87	107.20			
28	BE206D8.0949R1	Xq22	CH242-206D8			92.18	107.47			
29	BE386O15.1136R1	Xq22	CH242-386O15			92.27	107.86			
30	*IRS4.Y1*	Xq22	CH242-477D6			93.33	107.86		85.9	
31	MCSE313H19.0244	Xq22	CH242-313H19			93.72	108.38	67.5		
32	*ACSL4I3B259R*	Xq22	CH242-17O13		687	93.87	108.58			
33	***ACSL4I3B359M***	Xq22	CH242-17O13		687	**93.87**	108.58	67.9	87	
34	**MCSE231M24**	Xq22	CH242-231M24		682	**92.48**	109.41	69.8	88.1	
35	MCSE12P4.1041	Xq22	CH242-12P4			**92.78**	109.65	70.1		
36	MCSE12P4.0112	Xq22	CH242-12P4					71.2		
37	**MCST96O22**	Xq22	PigE-96O22		658	**92.99**	110.22	72.8	90.5	
38	**UMNP891**			2050	656			73.4	90.9	
39	BE95P6.0900R1	Xq22	CH242-95P6			94.38	110.48			
40	**MCSE347J6**	Xq22	CH242-347J6		646	94.52	110.63	74.5	92.3	
41	BE412O5B120R	Xq22	CH242-412O5			95.59	111.90			
42	BE80C18FB136W	Xq22	CH242-80C18			95.76		77		
43	BE504J7.0664Y1	Xq22	CH242-504J7			97.03				
44	**SW1522**	Xq22	CH242-408J11	2168	595	97.35	113.73	77.5	95.2	**55.4**
45	*HTR2CI3B151R*	Xq22	CH242-135K13			97.56	113.90		95	
46	BE371L5.0001Y1	Xq22	CH242-371L5			97.88	114.30			
47	BE185O8FB63S	Xq22	CH242-185O8			98.02	114.48	78.3		
48	BE219E21.0003M1	Xq22	CH242-219E21			98.16	114.79			
49	**UMNP93**			2214	540			79.1	96.5	
50	BE28B16.0529Y1	Xq23	CH242-28B16			99.34				
51	UMNP870	Xq23	CH242-141A6	2246	522	102.50	117.67			
52	**MCSI0244D12**			2261	506		118.15	81.6	98.8	
53	***SLC25A5I2B103DE***	Xq23	CH242-78C24			102.00	118.36	82.5	99.5	
54	UMNP1008	Xq23	CH242-458G8	2300	475	101.68	118.75			
55	**SW1943**	Xq24	CH242-105E5	2454	440	107.17	126.14	85	101.5	87.4
56	**SW1608**	Xq24	CH242-238J16	2650	304	112.30	132.31	98.4	114.1	101.9
57	SW707			2672	286				120.8	107.9
58	SW2137			2694	273					108.1
59	S0218	Xq25	CH242-1I19	2765	211	117.02		111.4		114.4
60	SW2588	Xq26	CH242-394H2	3117	0	125.93	150.01		159.7	128.4

^1^The most likely marker order was determined as the common order shared by most of maps.

^2^Markers placed on both INRA and JXAU genetic maps are indicated in bold letter, while gene-based markers are shown in italics.

^3^Pig clones were picked if their available sequences matched marker sequences through blast analysis at Sanger Center's website http://www.sanger.ac.uk.

^4^IMpRH1 (7000-rad) map was constructed by using Cathagene software.

^5^Positions of markers were determined by using the reference map of INRA2006 http://rhdev.toulouse.inra.fr/Do=Maps. This map was not oriented, and 0 cR corresponds the the last marker at the end of Xq arm.

^6^Locations of pig clones have been provided by UCSC Genome Browser http://pre.ensembl.org/Sus_scrofa_map/Location/Genome

^7^Placement information about homologous human sequences for porcine BAC end sequences is available at Sanger Center's website.

^8^Bold numbers indicate that the marker orders in two segments (*UMNP71-UMNP1218 *and *ACSL4I3B359M-MCST96O22*) on SSC-X clone map are inconsistent with those on the RH map, genetic maps and HSA-X physical map.

^9^Bold numbers indicate that the markers (*SW2476*, *SW259*, *SW1994*, *SW1426 *and *SW1522*) were in reverse order on the USDA-MARC map versus the current genetic maps or other maps.

An accurate genetic map is crucial for QTL analysis [13]. Notably, the present marker segment *SW259*-*SW1994*-*SW1426*-*SW1522 *observed on both INRA and JXAU genetic maps is reversed on the USDA-MARC genetic map [20] (Table 1), but is in accordance with both physical and RH maps, supporting the accuracy of our linkage map. Moreover, as mentioned previously by McCoard et al. [21], *SW2476 *was also placed wrongly on the USDA genetic map, which is revealed by its location on RH and physical maps (Table 1).

The INRA genetic map covered most of the chromosome length from *SW980 *(in Xp24) to *S0218 *(in Xq25). The ratio between the genetic and physical map is of 1.02 cM/Mb (111.4 cM for 109.3 Mb, Table 1). Two additional markers *SW949 *and *SW2588 *located at both ends in the Xp24/Yp pseudoautosomal region and Xq26 respectively were also mapped on JXAU families. The region from *SW949 *to *SW2588 *covers 126 Mb and 160 cM on JXAU genetic map (Table 1), thus corresponding to an overall average recombination rate of 1.27 cM/Mb.

**Figure 1:**
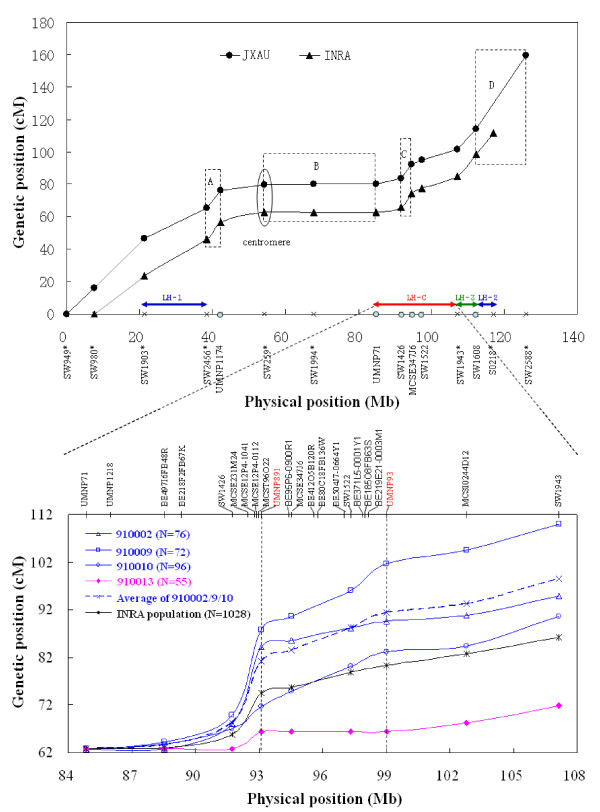
**The overall recombinational landscape of porcine X chromosome and intervals with individual heterogeneity of recombination**. The top part of this figure represents the genetic position of makers placed on both genetic maps of INRA and JXAU populations compare to the physical position of pig clone map. Markers with a star were used in the initial genotyping experiment. *SW259 *is located near the centromere. The slope of the curves provides an estimate of the local recombination rate. The patterns for the two populations are similar over the whole chromosome, except for the region from *SW980 *to *SW1903 *(gray lines). A (*SW2456-UMNP1174*), C (*SW1426-MCSE347J6*) and D (*SW1608*-*S*0218-*SW2588*) delimit three regions of high recombination rates; B (*SW259-SW1994-UMNP71*) is a recombination coldspot. Heterogeneity of recombination fraction among F_1 _females was observed for four intervals, including LH-C (*UMNP71-SW1943*), LH-1 (*SW1903-SW2456*), LH-2 (*SW1608-S0218*) and LH-3 (*SW1943-SW1608*). LH-1 and LH-2 were observed only in the INRA population, and LH-3 was specific to JXAU population, while LH-C was detected in both populations. The lower part presents a more detailed analysis of the recombination patterns in the LH-C interval among 4 F_1 _full-sisters from INRA population. Females 910002, 910009, 910010 who inherited the same maternal haplotype showed higher recombination rates compared with 910013, who inherited the other maternal haplotype, or compared with the average of the whole population. Number of meioses analysed for each female is presented between parentheses. LH-C could be further narrowed down to a sub-interval *UMNP891*-*UMNP93*, flanked by two vertical dash lines.

Regional variation in recombination rate along SSC-X is shown on Figure 1. Patterns of recombination appear very similar in the two populations. However, there are marked differences in the recombination rate between SSC-X regions. An extensive region (B) of very low recombination rate is especially striking (Figure 1). Between *SW259 *and *UMNP71*, and perhaps extending to *UMNP1218*, a fragment of more than 30 Mb represents only 0.1-0.45 cM (Table 1), which corresponds to a ratio of 0.015 cM/Mb. Only one recombination event out of 1027 meioses from the INRA population occurred in this region, between *SW259 *and *SW1994*. There were 6 recombination events out of 1338 meioses in the JXAU population, occurring in the 3 fragments flanked by markers *SW259*, *SW1994*, *UMNP71 *and *UMNP1218*.

Two regions (A and C; Figure 1) flanking the recombination coldspot, as well as the Xq telomeric region (D), show a rate of 2.8-3.3 cM/Mb, which was more than two-fold the value observed over the whole chromosome. The three regions may harbor recombination hotspots. Indeed, the fragment *MCSE231M24-MCST96O22 *within the region C presents an even higher ratio (3 cM for 510 kb ≈ 5.9 cM/Mb). In order to precisely map the potential recombination hotspot, we developed two additional microsatellite markers (*MCSE12P4.1041 *and *MCSE12P4.0112*) using the available partial sequence of one BAC clone (CH242-12P4; Genbank accession: CU618307) located in this region. As the fragment between the two markers is ~69 kb long and as it spans ~1.1 cM on INRA genetic map (11 recombination events out of 1060 meioses), the recombination rate increases to 15.9 cM/Mb in this segment.

### Variability of recombination rate between F1 females and populations

#### Identification of linkage heterogeneity in different regions of >10 cM on SSC-X

We initially detected heterogeneity in recombination fraction (θ) for large regions among F_1 _sows of the INRA family using Morton's likelihood ratio test [22] (Table 2). Significant differences among the 17 F_1 _sows were observed only in three noncontiguous intervals defined by *SW1903-SW2456 *(21.6 cM; *P *= 0.005), by *UMNP71-SW1943 *(24.2 cM; *P *= 0.002) and by *SW1608-S0218 *(12.8 cM; *P *= 0.038), denoted respectively LH-1, LH-C and LH-2 on Figure 1.

**Table 2:** Variability of recombination fraction (θ) in SSC-X regions of >10 cM among INRA F_1 _sows.

No. of sub-family^1^	F_1 _sows	Number of offspring	Marker intervals^2^					
			
			*SW980-SW1903*	*SW1903-SW2456*	*SW2456-SW259*	*UMNP71-SW1943*	*SW1943-SW1608*	*SW1608-S0218*
			
			θ	θ	θ	θ	θ	θ
1	910002	76	NA^3^	NA	0.184	**0.303^4^**	0.105	0.158
	910009	72	NA	NA	0.127	**0.417**	0.085	0.086
	910010	96	NA	NA	0.106	**0.271**	0.137	0.108
	910013	55	NA	NA	0.109	**0.091**	0.164	0.127
2	910014	83	0.228	0.316	0.241	0.253	0.146	0.064
	910016	38	0.184	0.342	0.132	0.211	0.132	0.184
	910020	69	0.206	0.232	0.159	0.159	0.159	0.059
3	910018	45	0.231	0.154	0.077	0.222	0.133	0.308
4	910069	62	0.213	0.295	0.161	0.145	0.097	0.210
	910071	68	0.246	0.123	0.154	0.206	0.132	0.091
	910072	63	0.206	0.111	0.175	0.206	0.143	0.143
	910074	83	0.241	0.190	0.177	0.120	0.181	0.157
5	910084	50	0.190	0.190	0.190	0.280	NA	NA
	910086	27	0.222	0.231	0.269	0.148	NA	NA
6	910095	62	0.177	0.113	0.210	0.242	0.081	0.161
	910096	42	0.171	0.250	0.175	0.262	0.024	0.073
	910097	37	0.243	0.081	0.027	0.189	0.189	0.054
								
Kosambi distance (cM)			22,86	21,63	16.58	24.20	13.17	12.80
Morton test			2,46	28.43	20.11	37.10	14.17	24.66
df			12	12	16	16	14	14
*P *value^5^			0.998	0.005	0.216	0.002	0.437	0.038

^1^Each sub-family consists of F_1 _full-sisters.

^2^The recombination coldspot *SW259-UMNP71 *was not considered.

^3^Not applicable because animal is not a double-heterozygote for both flanking markers.

^4^The θ of the F_1 _full-sisters within the first subfamily for the interval *UMNP71-SW1943 *are highlighted in bold because they showed the largest difference in θ (*P *= 0.0004).

^5^Probability for inter-individual variability of θ.

For the JXAU population, individual variability of θ among 59 F_1 _females was significant for the three intervals *SW949-SW980 *(16.0 cM; *P *= 0.037), *SW2456-SW259 *(14.7 cM; *P *= 0.042), *SW1943-SW1608 *(13.4 cM; *P *= 0.012; denoted LH-3 on Figure 1). However, only LH-C (*P *= 0.017) and LH-3 (*P *= 0.026) regions exhibit linkage heterogeneity when considering only the 40 F_1 _females having at least 20 offspring.

For all the regions of SSC-X, no significant difference was identified between the average rates calculated on INRA and JXAU families, except for the interval *SW980-SW1903 *(*P *= 0.005). This interval covers a distance of 23.7 cM in the INRA population in contrast to 30.6 cM in the JXAU population (Table 1), resulting in recombination rates of 1.7 and 2.2 cM/Mb respectively (Figure 1). This result agrees with previous observations showing that large-scale recombination pattern and hotspots tend to be conserved among human populations [23,24].

#### Fine mapping of the fragment showing linkage heterogeneity within the region UMNP71-SW1943

The interval *UMNP71-SW1943 *(LH-C) is of particular interest to us, because its linkage heterogeneity was highly significant in both populations and because many QTL have been mapped around this area http://www.animalgenome.org/cgi-bin/QTLdb/SS/draw_chromap?chromos=x. We tried to narrow down this region that showed linkage heterogeneity using additional markers. In the INRA population, only one sub-interval *UMNP891-UMNP93 *(5.9 cM; *P *= 0.001; Table 3) exhibited statistically significant linkage heterogeneity among F_1 _females, whereas other areas *UMNP71-SW1426 *(2.9 cM; *P *= 0.054), *SW1426-UMNP891 *(7.7 cM; *P *= 0.349) and *UMNP93-SW1943 *(5.9 cM; *P *= 0.458) did not. Therefore, the variation observed on the sub-interval *UMNP891-UMNP93 *explains most of the difference observed on the region as a whole.

**Table 3:** Variability of recombination fraction (θ) for the *UMNP891-UMNP93 *interval among INRA F_1 _female individuals, and among groups of females grouped according to their maternal and paternal haplotypes^1^.

No. of sub-family^2^	F_1 _sows	Paternal haplo-type	Maternal haplo-type	N	θ	F_1 _maternal haplo-type	N	θ	F_1 _paternal haplo-type	N	θ
1	910002	2413	1122	76	**0.053^3^**	1122	243	0.103	2413	574	0,059

	910009	2413	1122	72	**0.139**	1222	526	0.055	5113	235	0,055

	910010	2413	1122	95	**0.116**	3221	38	0.105	5413	141	0,043

	910013	2413	4311	55	**0**	4311	138	0.007	6323	78	0,090

2	910014	5113	1222	83	0.096	5221	83	0.012			

	910016	5113	3221	38	0.105						

	910020	5113	1222	68	0.015						

3	910018	5113	4311	46	0						

4	910069	2413	1222	62	0.032						

	910071	2413	1222	68	0.044						

	910072	2413	1222	63	0.048						

	910074	2413	5221	83	0.012						

5	910084	6323	1222	51	0.118						

	910086	6323	1222	27	0.037						

6	910095	5413	1222	62	0.048						

	910096	5413	1222	42	0.048						

	910097	5413	4311	37	0.027						

											

Kosambi distance (cM)					5.86			5.86			5.86

Morton test					39.24			23.61			1.96

Df					16			4			3

*P *value					0.0010			0.0001			0.5800

^1^In the region *UMNP891*-*UMNP93*, 4 microsatellite markers (*UMNP891*, *MCSE347J6*, *SW1522*, *UMNP93*) and 7 SNPs were genotyped. For simplicity, we indicated the haplotypes with only microsatellites alleles because they reveal all individual haplotypes formed by all markers analysed on these animals.

^2^Each sub-family consists of F_1 _full-sisters.

^3^The θ of the F_1 _full-sisters within the first subfamily are highlighted in bold because they showed the largest difference in θ (*P *= 0.0026).

Similarly, significant difference in θ for the interval *UMNP891-UMNP93 *(5.6 cM; *P *= 0.006) was also observed among F_1 _females from JXAU population. Unfortunately, in this population, *UMNP891 *was not informative for some F_1 _females and *UMNP93 *was not genotyped for all samples. Thus, the heterogeneity was tested and confirmed on a larger set of meioses using the two flanking markers *MCST96O22 *and *MCSI0244D12 *(*P *= 0.002). Linkage heterogeneity was not detected in other intervals *UMNP71-SW1426 *(3.4 cM; *P *= 0.204), *SW1426-MCST96O22 *(7.0 cM; *P *= 0.730) and *MCSI0244D12-SW1943 *(2.7 cM; *P *= 0.778). Relatively higher *P *values in these sub-intervals obtained therein could explain why linkage heterogeneity for the overall interval *UMNP71-SW1943 *was less significant in the JXAU population than in the INRA population.

#### Variation in recombination fraction for the interval UMNP891-UMNP93 within an INRA family consisting of 4 F1 full-sisters

The most significant difference in θ for the interval *UMNP891-UMNP93 *among F_1 _full-sisters was observed in the subfamily No.1 (*P *= 0.0026; Table 3). All F_1 _full-sisters inherited the same paternal X chromosome from LW boars whereas they inherited one or the other maternal X chromosome from MS sows. We noticed that in the subfamily No.1, three F_1 _full-sisters (910002/9/10) carrying the same maternal haplotype (1122) within the interval *UMNP891-UMNP93 *tended to have higher θ (5%, 14%, and 13%, respectively; Table 3). Among these three full-sisters, the relatively low recombination rate observed for 910002 in this interval may result from interference because a high crossover rate was observed in the neighboring region (Figure 1). In contrast, the last full-sister 910013 inherited the other maternal haplotype (4311) and had no recombination in this region at all. No other F_1 _females carried the haplotype 1122. However, 910013's relatives 910018 and 910097 who also carried the maternal haplotype 4311 also exhibited very low θ (Table 3). Based on the knowledge of parental relationship since the importation of Meishan animals in France and of the genotypes obtained on some key Meishan ancestors, we can show that the haplotype 4311 observed in the three females 910013, 910018 and 910097 is highly likely to represent an identical by descent (IBD) (See Additional Files 2 and 3 - Figures S1 and S2),.

### Impact of maternal and paternal haplotypes on recombination rate variation

Seventeen F_1 _females from the INRA population were grouped by their maternal (MS) or paternal (LW) haplotypes in the interval *UMNP891-UMNP93*, respectively (Table 3). The linkage heterogeneity is strongly significant among sets of F_1 _females grouped according to the Chinese haplotype inherited from their mother (*P *= 0.0001), whereas no heterogeneity is observed when F_1 _females are grouped according to the European haplotype inherited from their father (*P *= 0.580). The θ for haplotype 1122 and 4311 differed by more than fourteen-fold (10.3% vs. 0.7%). We indentified after genotyping additional SNPs, that the only one recombination (seen in a piglet of 910091) occurring in a 4311 haplotype happened at the end of segment between markers *BE371L5-0001Y1 *and *BE219E21-0003M1*. Thus globally, no recombination occurred in approximately 5 Mb in the haplotype 4311 in the 138 meiotic events. The most frequent maternal haplotype found in F_1 _females was 1222, with an average θ of 5.5%, a value equivalent to the average θ for all haplotypes.

Similar results for linkage heterogeneity were obtained from the JXAU population for the interval *MCST96O22-MCSI0244D12 *(Table 4). All JXAU F_1 _females were grouped into two paternal half-sib families according to only two WD boars (0F11 and 0F21) mated with ER sows. No significant difference (*P *= 0.414) in θ was found between these two groups of families. Within each of the two groups, when F_1 _females were also grouped according to the haplotype inherited from their Erhulian mother, a significant difference was put in evidence within the 0F21 family (*P *= 0.002) but not in the 0F11 family (*P *= 0.195). This latter result might be simply due to a smaller sample size for the 0F11 family. The θ for the same maternal haplotypes (except for haplotype 9512116 whose sample size was small) from different paternal families did not differ significantly (*P *> 0.05). Almost no common Chinese haplotype in this interval was shared between F_1 _females of INRA and JXAU populations.

**Table 4:** Variability of recombination fraction (θ) for the *MCST96O22-MCSI0244D12 *interval among JXAU F_1 _females grouped by their paternal and maternal haplotypes^1^.

JXAU paternal half-sib families (F_0 _♂ haplotype = F_1 _paternal haplotype)^2^	N	θ	F_1 _maternal haplotypes within 0F11 family^2^	N	θ	F_1 _maternal haplotypes within 0F21 family^2^	N	θ
0F11 {6 [5414] 12}	518	0.071	**0F11_7 [5325] 17**	95	0.042	0F21_1 [1116] 17	35	0.000

0F21 {2 [2514] 12}	775	0.084	**0F11_1 [5122] 35**	12	0.083	0F21_1 [1321] 17	60	0.167

			**0F11_1 [5321] 13**	64	0.047	**0F21_7 [5325] 17**	72	0.056

			**0F11_5 [1321] 14**	79	0.076	0F21_7 [5621] 14	24	0.042

			0F11_5 [1321] 17	137	0.095	0F21_1 [5121] 13	23	0.000

			0F11_5 [1325] 17	18	0.056	**0F21_1 [5122] 35**	156	0.051

			0F11_5 [5321] 14	56	0.107	0F21_1 [5225] 11	12	0.083

			0F11_5 [5325] 17	20	0.150	**0F21_1 [5321] 13**	133	0.120

			**0F11_9 [5121] 16**	37	0.000	0F21_1 [5341] 38	22	0.227

						0F21_5 [1125] 49	28	0.036

						**0F21_5 [1321] 14**	153	0.065

						0F21_5 [5121] 14	50	0.140

						**0F21_9 [5121] 16**	7	0.286

								

Kosambi distance (cM)		7.96			7.19			8.47

Morton test		0.67			11.12			30.59

df		1			8			12

*P *value		0.414			0.195			0.002

*P*_r _value^3^					0.090			0.006

^1^The haplotypes are formed by microsatellite markers (*MCST96O22*, *UMNP891*, *MCSE347J6*, *SW1522*, *UMNP93*, *UMNP870*, *MCSI0244D12*) within the interval of interest.

^2^Maternal haplotypes in bold are common to F_1 _females from both 0F11 and 0F21 families. The number between brackets represents the haplotype over the core *UMNP891-UMNP93 *interval.

^3^The *p*_r _values are determined after removing the haplotypes with <30 meioses.

## Discussion

### Comparative map

To date, the pig RH map - human comparative map is not available for X/Y chromosomes in the pig QTL database http://www.animalgenome.org/cgi-bin/QTLdb/SS/link_rh2hs?chromos=X, therefore the SNP sequence matches to the human genome cannot be easily aligned to the QTL map. Here, we provide the links between the pig RH map, the pig clone map and the human physical map. These will facilitate the search for candidate genes for traits of interest by fine comparison of the porcine regions with corresponding segments of human genome, and will enable to understand the evolution of these chromosomes. Comparison of the pig and human X chromosome maps revealed remarkable conservation of sequence order along the entire X chromosomes, including the location of the centromere. This is the same case for horse X chromosome [25], whereas some breakpoints and chromosomal rearrangements were found when comparing mouse and human or cow and human X chromosomes. (http://www.ensembl.org/Homo_sapiens/Location/Synteny?otherspecies=Bos_taurus&r=X%3A151175332-151275332; [26]).

### Regional variation in recombination rate

In the present study, we identified considerable variation in regional recombination along SSC-X. A large recombination coldspot adjacent to the centromere of X chromosome has been previously reported for human [27,28] and suggested for pig by alignment of the USDA linkage and cytogenetic maps (http://www.marc.usda.gov/genome/swine/htmls/Chromosomexy.html; [20,21]) This study not only confirms that this coldspot exists in pigs, but also estimates its extent (*SW259-UMNP1218*; ~31 Mb) and recombination fraction (<0.4 cM), which is likely to be longer and "colder" than the counterparts in other mammals. In humans, the coldspot is 17 Mb and 1 cM in size [28]. The coldspot can not be completely explained by a "centromere effect", as the centromere is only at one end of the coldspot. Shashi et al. [29] reported a three-generation human family with a large pericentric inversion of the X chromosome. Recombination was observed only at the telomeric regions Xp22 and Xq27-28, outside the inverted region, and fertility was not obviously affected in the carriers of this inversion. Whether there is a chromosomal inversion on SSC-X in European or Chinese pigs, leading to low recombination will need further investigation.

In rat and mouse, the X chromosome has lower recombination rate than the autosomal average and HSA-X has a rate very near the human genome-wide average [1], whereas we found that the average rate across SSC-X was a bit higher than the global level of the pig genome (~1.27 cM/Mb vs. ~0.92 cM/Mb for female-specific [19]). This might be attributable to several regions of higher recombination that would compensate for the large region of low recombination on SSC-X. Such possible regions mainly distribute at neighborhood sites of the coldspot and near telomeres. We fine mapped such a hotspot in the clone CH242-12P4 within the SSC-Xq22 region, with a rate as high as 15.9 cM/Mb. The smaller the interval examined, the greater the regional variation in recombination rate. On the HSA-X, 608 hotspots mapped within 5 kb [30]. Hotspots are not conserved among species [15,31].

### Inter-individual, inter-family and inter-population variation in recombination rate

We noticed two differences between the INRA and JXAU results. First, the genetic length of the interval *SW980-SW1903 *differed significantly between the two populations (23.7 vs. 30.6 cM). Second, some intervals (such as *SW1903-SW2456*) that showed significant linkage heterogeneity among individuals were population-specific.

On the other hand, there are strikingly consistent findings that the individual variability of recombination rate in the interval *UMNP891-UMNP93 *is significant in both populations. To our knowledge, the present study is the first to provide evidence of differences in fine-scale recombination rate among females, supporting the fact that genome-wide recombination rate varies substantially among women [13,14].

### Haplotype effect on recombination

Some early studies have documented that meiotic recombination in the MHC region is likely to depend on haplotypes [16,17]. Now, we found the distribution of crossovers in the interval *UMNP891-UMNP93 *also obeys this principle. Moreover, the recombination variation in this interval was only associated with the maternal haplotypes (Chinese pig haplotypes) rather than the paternal haplotypes (European pig haplotypes) of F_1 _females. Due to the structure of the two pedigrees used in this study, it is not possible to formally differentiate a haplotypic effect from a parental effect (maternal versus paternal). However, a haplotypic effect among the different Asian haplotypes seems the most plausible explanation for the findings. Such an effect could be explained by a simple global inversion of ~5 Mb in the haplotype 4311 or by differences in the sequence of the different haplotypes. Cytogenetic analysis will be further required, but we don't think that all differences in the recombination rate could be explained by a simple chromosomal fragment inversion as differences in the recombination rate are identified in both populations, whereas the Chinese haplotypes in segregating in both populations are completely different. We were not able to provide this cytogenetic evidence for the animals of this study as we did not froze cells to prepare metaphases. The differences in the recombination rate among haplotypes might be due to DNA sequence divergence in Chinese pigs, different chromatin structure, imprinting and/or their interactions. At present we cannot rule out any possibilities. The genetic basis of recombination variation has not been fully understood. For humans, specific DNA motifs and repeats are strongly associated with recombination rate, while there is no association between recombination rate and DNaseI hypersensitivity [32,33]. The evidence for a link between imprinting and recombination rate is currently weak [2,34]. The future availability of the porcine reference sequence will likely help us to understand the basis of this linkage heterogeneity.

### Consequence of variation in recombination rate for QTL mapping

Low recombination in almost one-fourth of the length of SSC-X is a serious problem for interpretation of QTL mapping results and fine mapping of these QTL, as many genes in the "cold" region could be associated with a quantitative trait. In fact, a noticeable clustering of QTL, especially of the "major" QTL, is observed near the centromere (http://www.animalgenome.org/cgi-bin/QTLdb/SS/draw_chromap?chromos=x; [6,35]). This might be attributable to the extraordinary high gene density per centiMorgan in the region with different genes with polygenic effect acting as a single strong QTL effect. This situation would be consistent with the conclusion made on humans by Boyle et al. [36]. In that case, if the causative mutations can not be identified, the haplotype block could be globally eliminated or selected in Marker Assisted Selection programs. For QTL mapped in a region of low recombination, haplotype analysis and association studies, combined with the careful comparison of QTL effects identified in different populations, might provide more valuable information than linkage analysis; yet identifying the causative mutations is unlikely to be achieved through these approaches [37]. Additional strategies, such as expression QTL (e-QTL) mapping, may enhance gene-mapping efforts.

The presence of inter-individual, inter-family and inter-population variation in recombination rate can also bias conclusions from genetic mapping studies. For QTL in the region where heterogeneity in recombination rate occurs, the estimation of position and effect of QTL are both altered by the differences between the average linkage map and the true recombination pattern of each F_1 _females, subfamilies or populations. Nevertheless, the consequences of individual variation need not always be negative [2]. It could lead to the identification of either chromosomal variation or modifier genes linked or unlinked to the interval under study, and perhaps yield new insight into the mechanism of mutation [8]. A general role of DNA repeats in mediating disease-causing recombination errors has been suggested [38]. If reduced recombination is a common result of sequence mismatch in the mutated region, this could even become a new positional mapping approach--that is, screening carriers for perturbed recombination [2].

## Conclusion

We provide the first published comparative map by integrating marker sequence positions of both pig and human chromosome X. The comparative map confirms the conservation of synteny between SSC-X and HSA-X, and will be valuable for selection of candidate genes for porcine QTL that map to SSC-X. Large differences in broad- and fine-scale recombination rate along SSC-X and between F_1 _females were revealed, which may cause unpredictable difficulties to precisely estimate the position and effect of individual causative gene. Recombination variation over the interval *UMNP891-UMNP93 *was associated with maternal haplotype of Asian origin of F_1 _females. A future comparison of sequences of these haplotypes will be very interesting to identify the cause of these variations

Globally, our results highlight the necessity of careful fine mapping of QTL identified in segregation in pig breeds on SSC-X relatively to coldspot, hotspot and LH regions identified in this study.

## Methods

### Animals

All animal experiments were conducted in accordance with European Communities Council Directive of 24 November 1986 (86/609/EEC) and the Guidelines for the Care and Use of Animal established by the Ministry of Science and Technology of P.R. China (1988). The structures of the INRA (LW × MS) and JXAU (WD × ER) populations have been described by Bidanel et al. [18] and Guo et al. [19], respectively. Briefly, for INRA population, 6 F_1 _males and 23 F_1 _females, the progeny of 6 LW boars and 6 MS sows, produced 530 F_2 _males and 573 F_2 _females. Six F_1 _females were culled early and were removed from the experiment. The 17 remaining sows were used to produce up to 13 litters, resulting in 1028 F_2 _piglets. Two of the 6 males were culled before the end of the experiment. Their females were reassigned to the four remaining males in order to produce additional full-sib families. For JXAU population, a total of 9 F1 males and 59 F1 females, the progeny of 2 WD boars and 17 ER sows, were randomly chosen to produce 967 F2 males and 945 F2 females from the first parity to the fourth parity in six separate batches. To obtain large full-sib families, each F1 sow was usually mated to the same sire during the different parities. In this study, a total of 1028 F_2 _animals of INRA and 1293 F_2 _animals of JXAU and their parents and grandparents were genotyped. The number of genotyped offspring of each F_1 _female from JXAU population varied from 5 to 49, with the mean of 22; whereas 17 F_1 _females from INRA population individually had at least 27 genotyped offspring and most of them had more than 50, which makes the estimation of variation in recombination rate among individuals more robust.

### Marker genotyping

Fifty-eight markers were used in this study (Table 1). Among them, 15 microsatellite markers with the caption of "SW" and *S0218 *were chosen from the USDA-MARC porcine reference map http://www.marc.usda.gov/, and 9 UMNP markers were selected from papers published by the University of Minnesota [39-41]. Single nucleotide polymorphisms (SNP) in the *SERPINA7 *gene and a 14-bp deletion mutation in *SLC25A5 *have been reported by Nonneman et al. [42] and Čepica et al. [43], respectively. The SNP in other three genes *ACSL4*, *IRS4 *and *HTR2C *are new markers. Moreover, we developed 18 additional SNP (named "BE...") and 11 microsatellites (named "MCSE...") based on the pig bacterial artificial chromosome (BAC) clone sequences and map http://pre.ensembl.org/Sus_scrofa/Info/Index. Primers were designed using Primer3 software http://frodo.wi.mit.edu/primer3/. Forward primers for most microsatellite markers were modified by adding an M13(-21) tail (5'-TGTAAAACGACGGCCAGT-3') to their 5' ends [44]. For fragments that do not contain a microsatellite, the possible polymorphism was firstly determined by high-resolution melting analysis on a LightCycler 480 (Roche), before confirmation by sequencing. The PCR profiles included an initial denaturation at 94°C for 5 min followed by 35-45 cycles of 94°C for 30 sec, annealing temperatures (50-60°C) for 30 sec and 72°C for 30 sec, with a final extension at 72°C. Except two new gene-based (*IRS4 *and *HTR2C*) SNP that were examined by SNaPshot (Applied Biosystems; Foster City, CA, USA), all currently developed SNP were genotyped by the PCR-RFLP method (Additional File 1 - Table S1). For microsatellites, PCR products were analyzed on an ABI PRISM 3130 or 3730 Sequencer and the genotypes were determined by performing allelic discrimination using GeneMapper 3.7 software (ABI, Foster City, USA). All genotypes were checked and stored using the GEMMA database https://www-lgc.toulouse.inra.fr/internet/index.php/Tools/Gemma.html.

### RH mapping

Markers were mapped on the 7000-rad IMpRH panel [45] or 12000-rad IMNpRH2 panel (for a few highly linked markers) [46] according to the INRA protocols. Data were analyzed for two-point and multipoint linkage with the IMpRH mapping tool and submitted to the IMpRH web server (http://imprh.toulouse.inra.fr/; [47]). Carthagene software (http://www.inra.fr/bia/T/CarthaGene/; [48]) was also used to estimate multipoint marker distance and order using all public markers on the X chromosome in the IMpRH sever and those developed in this study, in order to compare the former map automatically built by the server.

### Linkage analyses

The female-specific linkage maps for INRA and JXAU were calculated using CRIMAP version 2.4 [49] as described by Rohrer et al. [20] where TWOPOINT analyses were used to indicate the chromosome linkage group and the BUILD, ALL, FLIPS options were used to determine the most likely multipoint position of each marker.

### Placement of markers on pig clone map and estimates ofrecombination along SSC-X

The full sequences of markers were analysed using BLAST http://www.sanger.ac.uk/cgi-bin/blast/submitblast/s_scrofa for identification of clones used in pig genome assemblies. Hit locations of these clones on Human chromosome X (HSA-X) have been presented on the Sanger web http://www.sanger.ac.uk/cgi-bin/Projects/S_scrofa/WebFPCreport.cgi. The ratio between genetic and physical distances between each pair of adjacent markers was calculated by simply dividing the distances between the markers on the genetic map (in Kosambi centimorgans, cM) by the distance between the markers on the BAC clone map (in megabases, Mb).

### Statistical analyses

Recombination counts and total counts over a specific marker interval for each F_1 _female that was doubly heterozygous for the adjacent markers, and the haplotypes of all F_1 _females were exported from the GEMMA database https://www-lgc.toulouse.inra.fr/internet/index.php/Tools/Gemma.html. The Morton's likelihood ratio test [22] was applied to test the individual variability of recombination fraction as described by Simianer et al. [9].

## Abbreviations

INRA: National Institute of Agricultural Research in France; JXAU: Jiangxi Agricultural University (JXAU); LW: large white pigs; MS: Meishan pigs; WD: white Duroc pigs; SSC-X: porcine chromosome X; HSA-X: human chromosome X; MHC: major histocompatibility complex; QTL: quantitative trait locus; RH: radiation hybrid; BAC: bacterial artificial chromosome; cR: centi Ray; cM: centi Morgan; Mb: megabase; θ: recombination fraction; IMpRH: the INRA-University of Minnesota porcine radiation hybrid panel; IMNpRH2: the INRA Minnesota Nevada porcine Radiation Hybrid panel 2.

LH-1, LH-2, LH3 and LH-C respectively represent the interval *SW1903-SW245*, *SW1608-S0218*, *SW1943-SW1608 *and *UMNP71-SW1943*, which showed linkage heterogeneity among F_1 _females from single or both pig resource populations.

## Authors' contributions

JM performed the genotyping for INRA population and RH panel, analyzed the data and drafted the manuscript. NI assisted in performing the experiments and validated the data. YD, WH, BG performed the genotyping for JXAU population. JR participates in the design. LH and DM co-supervised the work. DM conceived the study and finalized the manuscript. All authors have read and approved the final manuscript.

## Supplementary Material

Additional file 1 **Table S1**. Information about the 33 markers developed in this study. Click here for file

Additional file 2 **Figure S1**. Haplotypes carried by the 6 F_0 _Meishan females. Click here for file

Additional file 3 **Figure S2**. Pedigree structure of some F_1 _animals' parents in different generations of Meishan pigs bred at INRA to determine if the haplotype 4311 associated with low recombination found in 3 F_1 _sows is IBD or IBS. Click here for file
