# Supplementary material for: Recombinational landscape of porcine X chromosome and individual variation in female meiotic recombination associated with haplotypes of Chinese pigs
Source: BMC Genomics. 2010 Mar 9;11:159. doi: 10.1186/1471-2164-11-159 (PMC2850356; doi:10.1186/1471-2164-11-159)
Supplement: Additional file 1 — Table S1. Information about the 33 markers developed in this study. [file 1471-2164-11-159-S1.DOC]

**Additional File 1**

**Table S1 - Information about the 33 markers developed in this study**

| Marker^1^ | PCR fragment size | Restriction enzyme | Mutation | PCR annealing temperature | Sequence Up^2^ | Sequence Down |
| --- | --- | --- | --- | --- | --- | --- |
| *ACSL4I3B259R* | 207 | Tsp509I | G>A | 56 | AGCAAACAAACACTGGCA | TCATCTGTGCCATTATGCCTAC |
| *ACSL4I3B359M* | 207 | MspI | A>C | 56 | AGCAAACAAACACTGGCA | TCATCTGTGCCATTATGCCTAC |
| BE102J23.0003R1 | 164 | MspI | A>G | 55 | GAATGGAAAGGATGGAAGAATCA | TCCCATATTCCTCATCTGTGTA |
| BE145J20.0597R1 | 137 | HpyCH4V | A>G | 55 | GGAACTCTGGTCAGTCAATGT | CTCAGTTATCTGATGGATTCACCT |
| BE151D17.0014Y1 | 120 | StuI | C>T | 55 | CACTCTGTGAGATTTGGGAATAC | CCCTTCTGCCAGCTTCCTAA |
| BE185O8FB63S | 259 | NlaIII | C>G | 58 | TCTGCTGATGGAGGAAAGTG | CTGAACTTCTATGAATGACATGGTAAC |
| BE206D8.0949R1 | 150 | MseI | A>G | 55 | GATTTGATACAGAATTTTCTCTTGTGTT | TGGCTGAAGAGAGGACGA |
| BE218F2FB67K | 175 | BsaJI | G>T | 58 | TGGCTTAGGAGTTGGACTTGATA | TGAACCGCCTACAAGCAC |
| BE219E21.0003M1 | 144 | DdeI | A>C | 55 | TGTTTCAGCCTCTCACAGTC | TGAAGTTCATAGAACAGGGTCA |
| BE276J1FB107R | 176 | HincII | A>G | 58 | TGTTTCCAGCAATCAAATCCG | GCCGATGGCTAGTGTATAATG |
| BE28B16.0529Y1 | 141 | AciI | C>T | 55 | GGGTTCCTACAGGGTTCCTTAAA | AATGGTATGGAATAGAGTTCAAGTG |
| BE32D24.0584R1 | 144 | BccI | A>G | 55 | GGATGACTGAAGAATAATCAGGTG | TTAGTGCAGATAAACCGTGCT |
| BE371L5.0001Y1 | 138 | RsaI | C>T | 55 | ACGGGAACTCCAAGAATATAAC | TTGCCAATAGAAACTGAGCTG |
| BE386O15.1136R1 | 150 | NlaIII | A>G | 55 | GATATCATACGGTATTTGTCTTTCTCT | TTTATTCAGTTGCCCGTCACAT |
| BE412O5B120R | 211 | Tsp45I | A>G | 58 | TGTCCCTGGAGCAGTGT | AGCTAAGGTCAGAGAGCG |
| BE497I6FB48R | 203 | BsrI | A>G | 58 | GCAACTTACACCACAGCTC | CTTCAACTTGGGAAAGCAACAT |
| BE504J7.0664Y1 | 119 | BsrI | C>T | 55 | AAATGTGAAATTGATGTATATTAGCGTT | TTTCTTAATTCAAAGTAGTGCTTTCTG |
| BE80C18FB136W | 136 | ApoI | A>T | 58 | CAGCAGCAGCTCCAATTAG | GAACCATTTATGTACTGTTGTGTTT |
| BE8B11.0679Y1 | 121 | RsaI | C>T | 55 | AGTTATAGCTCTGATGGTCGTG | TTCTCTCCTCTCCCTTTCTTTC |
| BE95P6.0900R1 | 137 | BsmI | A>G | 55 | GCTCTACTTCCAGTGGCATTTA | ATTTCCATTGCAGTCTGCTT |
| *HTR2CI3B151R* | 292 |  | A>G | 56 | ATGAGACTCCTGTGGGCTT | CATCTTGCTTACCTCTACTAG |
| *IRS4.Y1* | 266 |  | T>C | 56 | CACCCAGTGAGCCCTTGCC | CATATAGTCGCCTCCGTTTTCT |
| MCSE12P4.0112 | 172-212 |  | (CA)n | 58 | gttttcccagtcacgacgttgAGAAGGGAAACGTGAGACATA | CCCAGAGCAAATAGAAGAGG |
| MCSE12P4.1041 | 125-152 |  | (GT)n | 55 | gttttcccagtcacgacgttgTGGAATGTGTGAGTTTGTGG | GATCAAAGCATAGACTGAAGTAAGG |
| MCSE231M24 | 284-300 |  | (CA)n | 58 | gttttcccagtcacgacgttgTGAATCATGTGCTAGGCTTACATTAT | AGTCAATCAGTTAAATCCAAACTGT |
| MCSE313H19.0244 | 158-174 |  | (CA)n | 58 | gttttcccagtcacgacgttgTCTTGGGCTTCCTCACTTTAC | CTGGAAATCAAGGGAAAGAGAAT |
| MCSE347J6 | 157-174 |  | (CA)n | 58 | gttttcccagtcacgacgttgTTTCTGTACCCTCACCCATC | CATGGAAGTTTCACTCCAAACC |
| MCSE3F14 | 182-189 |  | (GT)n | 55 | gttttcccagtcacgacgttgCTTTCCCTAATGGAAGTGTCAG | AGCATTTTGTGTTTGCTCCA |
| MCSE58H4 | 205-252 |  | (TA)n | 55 | gttttcccagtcacgacgttgGCTTGGCTGAAATGAGAAAGG | CCTTTGATGTCTGCTTTTGG |
| MCSE65L7 | 353-357 |  | (AC)n | 58 | gttttcccagtcacgacgttgGCAATCACTGGACTGAGCCTA | TCTGCCCTAAACTTGTTGTTCTT |
| MCSI0244D12 | 210-264 |  | (AG)n | 60 | gttttcccagtcacgacgttgGCCAAGCACAGAGGCTTTAG | TGCAACACTCAGTGGAAAGG |
| MCST2J13 | 178-194 |  | (GT)n | 55 | gttttcccagtcacgacgttgGCATGCAAGCTTAGTGCAAA | TCCCTCCCCAACATACACAT |
| MCST96O22 | 226-256 |  | (AT)n | 58 | gttttcccagtcacgacgttgCAGGTATACAGCAAAGCAATTCA | GGTCCCTGTGCAAGGATG |

^1^Four gene-based SNP (*ACSL4I3B259R*, *ACSL4I3B359M, HTR2CI3B151R* and *IRS4.Y1*) are shown in italic text. For *HTR2CI3B151R* and *IRS4.Y*, their SNaPshot single base extension primer sequences are as follows:

*HTR2CI3B151R* : CTATTGTAAATTAGTAAATAAAGTAATGGTATTGGG; *IRS4.Y1*: CGCAGGTCCAGGAGAGCGGTTTCCGTGCCAGCCAGC.

^2^The forward primer for microsatellite markers contains universal M13 adaptor (5’-gttttcccagtcacgacgttg-3’).
