# Supplementary material for: Recombinational landscape of porcine X chromosome and individual variation in female meiotic recombination associated with haplotypes of Chinese pigs
Source: BMC Genomics. 2010 Mar 9;11:159. doi: 10.1186/1471-2164-11-159 (PMC2850356; doi:10.1186/1471-2164-11-159)

**Additional File 2**

**Figure S1. Haplotypes carried by the 6 F_0_ Meishan females.**

The haplotype patterns in the region *SW2456*-*SW1943* can be split into different sub-intervals. **(a)** All animals shared similar haplotype over the interval *SW1994*-*BE218F2FB67K*, occupying the most part of a recombination coldspot. Only one female 890690 was heterozygous for one marker (*MCSE58H4)* within the interval. **(b)** Five very distinct haplotypes (a-e) were found over the region *SW1426*-*UMNP891*, in which each of the four markers *SW1246*, *MCSE313H19-0244*, *MCST96O22* and *UMNP891* had 4 alleles. An additional (or a 6^th^) haplotype “f”, differing from the haplotype “d” by one marker (*MCSE12P4-0112*), is identified in female 890769. At that step we cannot determine whether haplotypes drawn in a given color are identical by decent (IBD) or identical by state (IBS). **(c)** In the region of linkage heterogeneity, we see that the haplotype “a” in pink shared by F_0_ females 890738, 8906090 and 890768 is extended in the region *MCSE247J6*-*MCSI0244D12*.


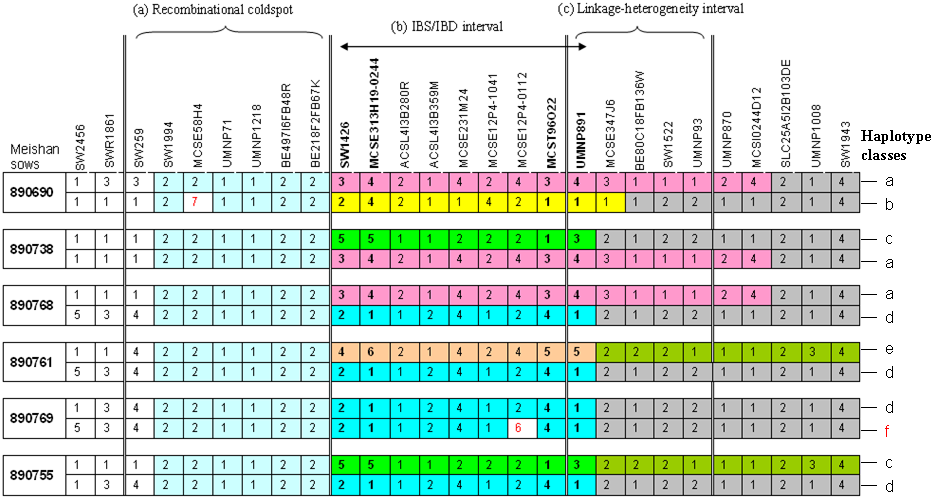

Supplement: Additional file 2 — Figure S1. Haplotypes carried by the 6 F0 Meishan females. [file 1471-2164-11-159-S2.DOC]
