# Supplementary material for: Recombinational landscape of porcine X chromosome and individual variation in female meiotic recombination associated with haplotypes of Chinese pigs
Source: BMC Genomics. 2010 Mar 9;11:159. doi: 10.1186/1471-2164-11-159 (PMC2850356; doi:10.1186/1471-2164-11-159)

**Additional File 3**

**Figure S2. Pedigree structure of some F_1_ animals’ parents in different generations of Meishan pigs bred at INRA to determine if the haplotype 4311 associated with low recombination found in 3 F_1_ sows is IBD or IBS.**

On the drawing, the names of F_0_ Large White males are underlined. F_0_ Meishan females are indicated in purple letters. On the pedigree, a red line links a mother to its offspring and a black line links a father to its offspring.

All Meishan animals from INRA herd derived from 5 founder boars and 2 founder sows (indicated in blue letters). A total of 9 X-chromosomes were thus in segregation. However, at least 2 X-chromosomes carried by two founder boars 810197 and 820370 (shown in blue and italic letters) were lost in the pedigree, as none of their daughters were used to produce subsequent generations. Therefore, in INRA Meishan animals, a maximal number of 7 different X chromosomes can be found. We have detected that in the linkage-heterogeneity region *UMNP891*-*UMNP93*, three F_1_ relatives 910013, 910018 and 910097 inherited the same maternal haplotype (4311 = part of the haplotype “a” on the supplementary fig. 1) from different Meishan females (F_0_). In order to know if this haplotype is IBD and not only IBS, we genotyped 8 microsatellite markers covering the region *SW1426*-*MCSI0244D12* on 14 ancestors Meishan (indicated with bold letters) for which DNA samples are available.

Based on known genotypes we followed the “a” haplotype (4311) in the previous generations. If a parent could not transfer the haplotype to child, the link line between them was marked with a cross. Animals that were not genotyped but which necessarily carried the “a” haplotype are indicated with a star. Here, we could deduce that 910013 necessarily received the 4311 haplotype from 810060 (indicated with a green dashed box), and 910018 and 910097 respectively had 50% and 25% probability to inherit it from this animal, too.

In Figure S1, we show that in region (b) we found 6 different haplotypes in F_0_ females, demonstrating that 6 X chromosomes of founders are ever represented in the 6 F_0_ females. Moreover, we found an additional haplotype, denoted “g” (not shown in detail) in male ancestor 840193 (and also in female 840194). Having thus identified the 7 different haplotypes from 7 founding chromosomes, we can be sure that in this interval all identical haplotypes are IBD and not only IBS. By extension in the flanking interval, it is highly likely that the extended haplotypes shared by females 890738, 890690 and 890768 in LH region (and transmitted to their daughters 910013, 910018 and 910097) are also IBD. In addition it is highly likely that these 3 F_1_ females all received this haplotype from their common ancestor 810060.


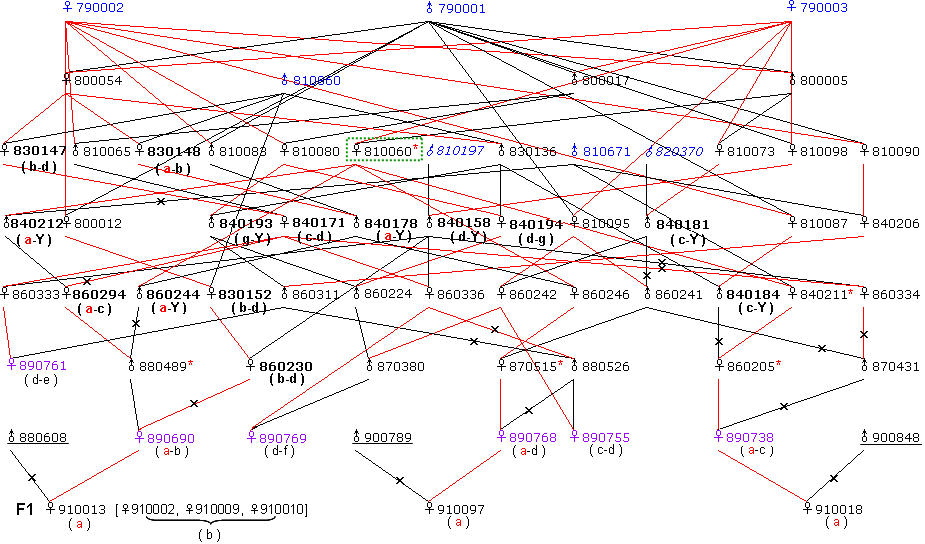

Supplement: Additional file 3 — Figure S2. Pedigree structure of some F1 animals' parents in different generations of Meishan pigs bred at INRA to determine if the haplotype 4311 associated with low recombination found in 3 F1 sows is IBD or IBS. [file 1471-2164-11-159-S3.DOC]
